# Texas State Conference of Charities and Correction

**Published:** 1914-12

**Authors:** 


### Texas State Conference of Charities and Correction.

The Texas State Conference of Charities and Correction is affiliated with the National Conference and has for its high aim the amelioration of suffering and the betterment of man's social condition. It seeks to uplift his community life and the abolishment of crime and poverty; the creation of a social atmosphere which would induce a right course of conduct. In this respect, like the above mentioned Congress, it has to do with environment also, but its scope is wider, in that it contemplates the State's duty in providing the agencies for social betterment. The Mothers' Congress is specialized work; the Conference of Charities and Correction is generalized work.

Among the most important subjects discussed and endorsed at its session last month for immediate action was the establishment of a farm colony for the treatment of professional vagrants and alcoholic habituates; institutions for the care and treatment of feeble-minded; compulsory education; a State bureau of charities; county hospitals for the care of the sick; psychopathic laboratories for the study of the psychoses; marriage regulation for preventing venereal diseases and other measures of a sociological nature. Even the "idle rich" came in for a touch up, and importance was given to providing occupation for the unemployed.

But what of all this? What of this vast expenditure of money and energy? What of the great tax of time on the part of the provident few? It is important, it is necessary, it is self-preservation. But why not carry our good purposes and endeavors still further, get at the root of the evil, and stop the flow? What would you think of a State bonding itself for a huge sum of money to meet necessary emergencies without providing a sinking fund by which this obligation could be met? You very well know there would come a time when all the State's resources would be exhausted and she would have to fall as a result of this lack of foresight. Just so with this tremendous problem of social betterment, money and energy spent in amelioration and no provision made for prevention must end in a complete failure, as history has repeated time and time again. Why not start that sinking fund—that preventive measure that would lessen the burden from year to year and eventually wipe it out? This is the mission of eugenics. Can we not join forces and lessen each other's burdens to the full fruition of our high purpose? As the solution of this problem is a question of education wholly, perhaps the speediest way to impart this knowledge would be along the suggestive lines pursued by such organizations as this. We, therefore, appeal to every eugenist and social worker to enlist in the cause of the Conference.

